# High flow nasal therapy versus noninvasive ventilation for AECOPD with acute hypercapnic respiratory failure: a meta-analysis of randomized controlled trials

**DOI:** 10.1186/s13613-025-01480-w

**Published:** 2025-05-14

**Authors:** Jinlv Qin, Guizuo Wang, Yixing Liao, Wenli Shang, Dong Han

**Affiliations:** 1 Radioimmunoassay Center, Shaanxi Provincial People’s Hospital, No. 256, West Youyi Road, Xi’an, 710068 Shaanxi China; 2 Department of Respiratory and Critical Care Medicine, Shaanxi Provincial People’s Hospital, No. 256, West Youyi Road, Xi’an, 710068 Shaanxi China; 3 Department of Critical Care Medicine, The First Affiliated Hospital, Zhejiang University School of Medicine, Hangzhou, 310003 Zhejiang China

**Keywords:** HFNC, NIV, COPD, Mortality

## Abstract

**Background:**

Guidelines recommend the use of noninvasive ventilation (NIV) and high-flow nasal cannula (HFNC) in patients with chronic obstructive pulmonary disease (COPD) and hypercapnic acute respiratory failure (ARF). It is unclear whether HFNC is noninferior to NIV in terms of the rate of tracheal intubation or mortality. This meta-analysis aimed to compare the efficacy of HFNC and NIV in patients with AECOPD and hypercapnic ARF.

**Methods:**

A systematic search was made of PubMed, Embase, Cochrane Library, and clinicaltrials.gov, without language restrictions. Randomized controlled trials (RCTs) on treatment of hypercapnic AECOPD with HFNC, compared with NIV, were reviewed. Estimated effects of included studies were pooled as risk ratios (RRs), with 95% confidence intervals (CIs).

**Results:**

Four RCTs (enrolling 486 patients) met the inclusion criteria. There was no statistically significant difference in all-cause mortality (RR 0.97, 95% CI 0.56 to 1.68), and intubation rate (RR 1.67, 95% CI 0.99 to 2.83) between the two groups. The treatment switch rate (RR 2.60, 95% CI 1.54 to 4.38) and treatment failure rate (RR 1.64, 95% CI 1.04 to 2.60) were significantly lower in NIV groups.

**Conclusions:**

Compared with NIV, HFNC was not associated with increased mortality and intubation rate. More patients receiving HFNC oxygen therapy experienced treatment failure and switched to NIV, which may mask the fact that HFNC is inferior to NIV in patients with AECOPD and hypercapnic ARF.

**Supplementary Information:**

The online version contains supplementary material available at 10.1186/s13613-025-01480-w.

## Introduction

Chronic obstructive pulmonary disease (COPD) is a common chronic respiratory disease that causes a gradual worsening of symptoms [1]. COPD can lead to acute exacerbations, which are characterized by the worsening of respiratory symptoms and the hypercapnic acute-on-chronic respiratory failure [2]. Patients with acute exacerbation of chronic obstructive pulmonary disease (AECOPD) who require hospitalization have a 6% risk of in-hospital mortality. Official guidelines of European Respiratory Society (ERS) and American Thoracic Society (ATS) recommend noninvasive ventilation (NIV), as first choice, for patients with COPD and acute hypercapnic acidotic respiratory failure (pH ≤ 7.35), including those requiring tracheal intubation and mechanical ventilation, unless the patient’s condition deteriorates immediately [3]. The application of high-flow nasal cannula (HFNC) in AECOPD has a physiological rationale (i.e., oxygenation, positive pressure, and reduced dead space) [4]. HFNC can wash out CO_2_ from the pharyngeal dead space (which accounts for approximately 30% of the total anatomical dead space) [5], and this is of great significance for patients with an increased ratio of dead space to tidal volume. HFNC generates a small amount of pharyngeal pressure (up to 8 cmH_2_O) during exhalation, and this pressure drops to zero during inhalation. This effect is similar to pursed-lip breathing, which is used to reduce the respiratory rate, prolong the expiratory time, and alleviate expiratory airflow limitation and dynamic pulmonary hyperinflation [5]. A meta-analysis [6] of patients with acute hypercapnic respiratory failure showed that, compared with NIV, HFNC demonstrated similar effects in improving arterial blood gas (ABG). Moreover, HFNC was more comfortable and resulted in fewer adverse events. Coupled with its ease of use and patient comfort, it has become an alternative to NIV for patients with hypercapnic acute respiratory failure (ARF) accompanied by respiratory acidosis [7]. However, its role in patients with COPD who present with hypercapnic ARF has not been fully established.

Therefore, the aim of this study was to conduct a meta-analysis of RCTs to determine whether HFNC is noninferior to NIV in patients with AECOPD and hypercapnic ARF.

## Methods

### Data sources and search strategy

This meta-analysis was based on the Preferred Reporting Items for Systematic Reviews and Meta-Analyses (PRISMA) statement [8]. The protocol was previously registered in December 2024 in the PROSPERO database (Review register: CRD42024631973). The PubMed, Embase, Cochrane Library, and clinicaltrials.gov were searched for studies from each database’s inception to December 27, 2024.

### Study selection

To be eligible for inclusion in the meta-analysis studies had to meet the following criteria: (a) inclusion of AECOPD patients presenting with hypercapnic ARF (pH ≤ 7.35, arterial carbon dioxide partial pressure (PaCO_2_) > 45 mmHg); (b) use of a randomized controlled design to make a comparison of HFNC with NIV; and (c) outcomes include mortality or the rate of tracheal intubation. The search strings used for PubMed were (“high flow” OR “HFNC”) AND (“chronic obstructive pulmonary disease” OR “COPD”) AND (“hypercapnic” OR “acidosis” OR “hypercapnia” OR “acidotic”). The reference lists of any relevant review articles were also screened to identify studies that might have been missed in this search. No language restrictions were applied to our study selection process. The full search strategies for all databases are provided in Table S1.

### Data extraction and quality assessment

Two reviewers independently screened articles according to the inclusion criteria. The reviewers compared selected studies and differences were resolved by consensus. Data tables were used to collect all relevant data from texts, tables and figures of each included trial, including author, year of publication or last update posted, patient number and age, baseline body mass index (BMI), arterial pH, PaCO_2_, arterial oxygen partial pressure (PaO_2_)/fraction of inspired oxygen (FiO_2_), and outcomes such as all-cause death, tracheal intubation, treatment failure (defined as the requirement for intubation and invasive ventilation, or a switch to the other treatment group), and treatment switch (switch from HFNC to NIV, or switch from NIV to HFNC).

### Risk of bias of included trials

Two reviewers independently assessed the risk of bias using the Cochrane collaboration risk of bias tool for RCTs [9].

### Data synthesis and statistical analysis

Meta-analyses were conducted where applicable; otherwise, outcomes were presented in narrative form. Data were analyzed using the RevMan Version 5.1 (The Cochrane Collaboration). Next, risk ratios (RRs) for discontinuous outcomes, with corresponding 95% confidence intervals (CIs) were computed for individual trials. Chi-squared and Higgins I^2^ tests were used to assess heterogeneity among included trials. If significant heterogeneity (p ≤ 0.10 for Chi-squared test results or I^2^ ≥ 50%) was obtained, we used a random-effects model, otherwise a fixed-effects model was used. And a P value < 0.05 was taken to indicate statistical significance.

## Results

### Study selection and characteristics

**Fig. 1:**
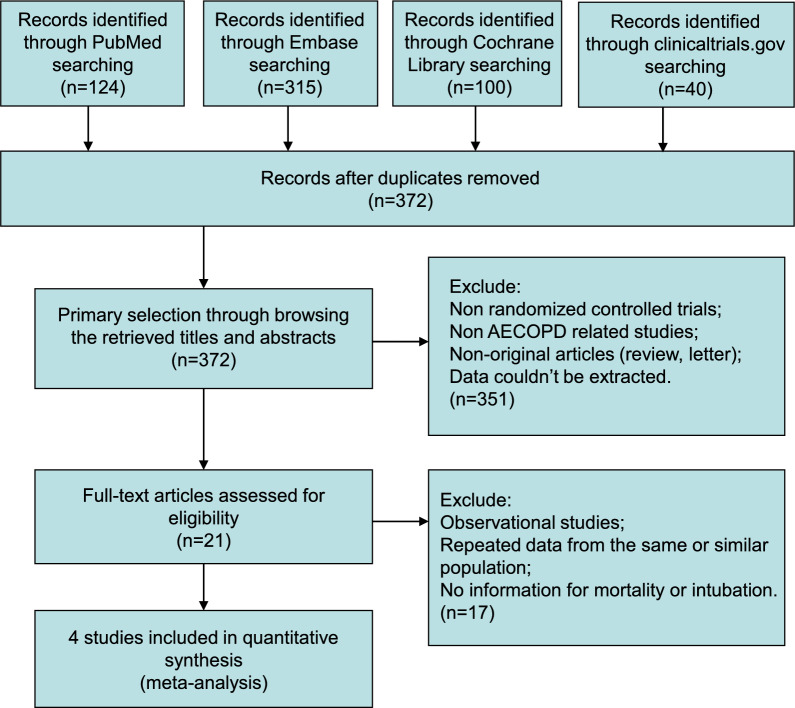
Flow chart for selection of studies

**Fig. 2:**
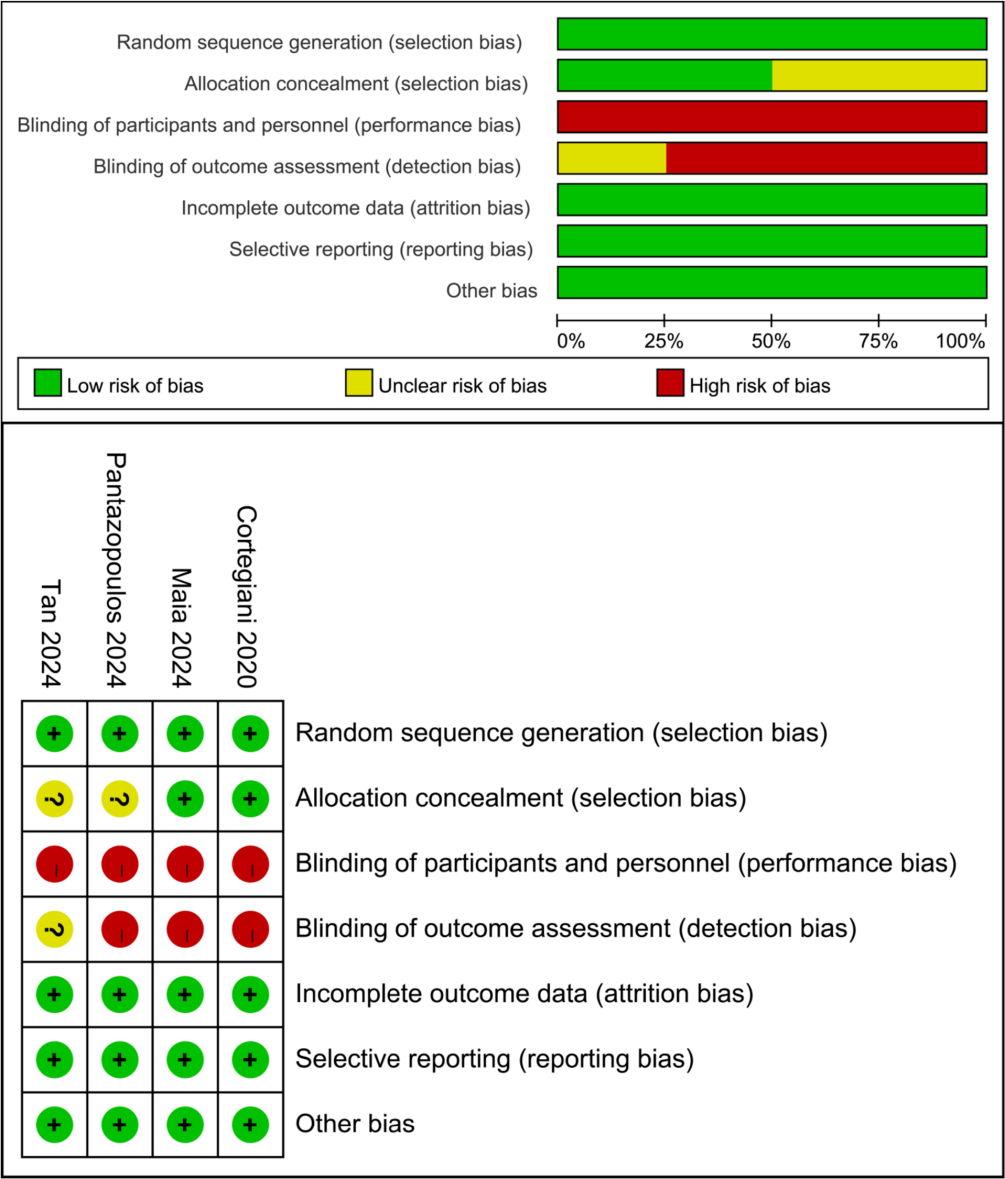
Assessment on risk of bias for included RCTs

Of 579 studies recognized by the initial search, 21 were retrieved for more detailed assessment, and 4 trials [10–13] were included in this meta-analysis (Fig. 1). Baseline characteristics of trials included in this meta-analysis are shown in Table 1. A total of 486 patients were included: 239 assigned to the HFNC therapy groups and 247 to the NIV groups. The risk of bias results are summarized in Fig. 2.

**Table 1:** Baseline characteristics of trials included in meta-analysis

Study (Refs. #)	Year	ABG	Device, mode	n	Age, years (SD)	Male, %	BMI, kg/m^2^ (SD)	PaCO_2_, mmHg (SD)	Arterial pH (SD)	PaO_2_/FiO_2_ (SD)
Cortegiani [10]	2020	pH 7.25–7.35, PaCO_2_ ≥ 55 mmHg	HFNC, Airvo-2 or Optiflow NIV, BiPAP	40 39	74 (13) 77 (12)	53 49	30.5 (8.7) 26.7 (5.5)	73.7 (12.8) 72.0 (13.0)	7.30 (0.03) 7.29 (0.03)	203.2 (45.5) 222.4 (71.0)
Maia [11]	2024	pH < 7.35, PaCO_2_ > 45 mmHg	HFNC, Airvo-2 NIV, BiPAP	35 42	74 (10) 71 (11)	57 38	NR NR	55 (46–73)* 64 (51–73)*	7.32 (7.28–7.34)* 7.30 (7.24–7.36)*	189 (143–290)* 209 (163–308)*
Pantazopoulos [12]	2024	pH 7.25–7.35, PaCO2 > 45 mmHg	HFNC, Airvo-2 NIV, BiPAP	51 54	72 (9) 73 (10)	67 76	25.0 (5.1) 25.5 (5.2)	61 (10) 65 (13)	7.30 (0.03) 7.29 (0.05)	237.8 (58.3) 238.1 (43.2)
Tan [13]	2024	pH 7.25–7.35, PaCO_2_ ≥ 50 mmHg	HFNC, Airvo-2 NIV, BiPAP, V60 or Vision	113 112	73 (65–78)* 69 (63–76)*	63 55	NR NR	63 (59–68)* 61 (58–65)*	7.31 (7.29–7.33)* 7.30 (7.28–7.32)*	175 (167–199)* 184 (167–202)*

*ABG* arterial blood gas analysis, *BiPAP* bi-level positive airway pressure, *BMI* body mass index, *FiO*_*2*_ fraction of inspired oxygen, *HFNC* high-flow nasal cannula, *NIV* noninvasive ventilation, *NR* not reported, *PaCO*_*2*_ arterial carbon dioxide partial pressure, *PaO*_*2*_ arterial oxygen partial pressure, *SD* standard deviation. *values are median and interquartile range

### All-cause death

**Fig. 3:**
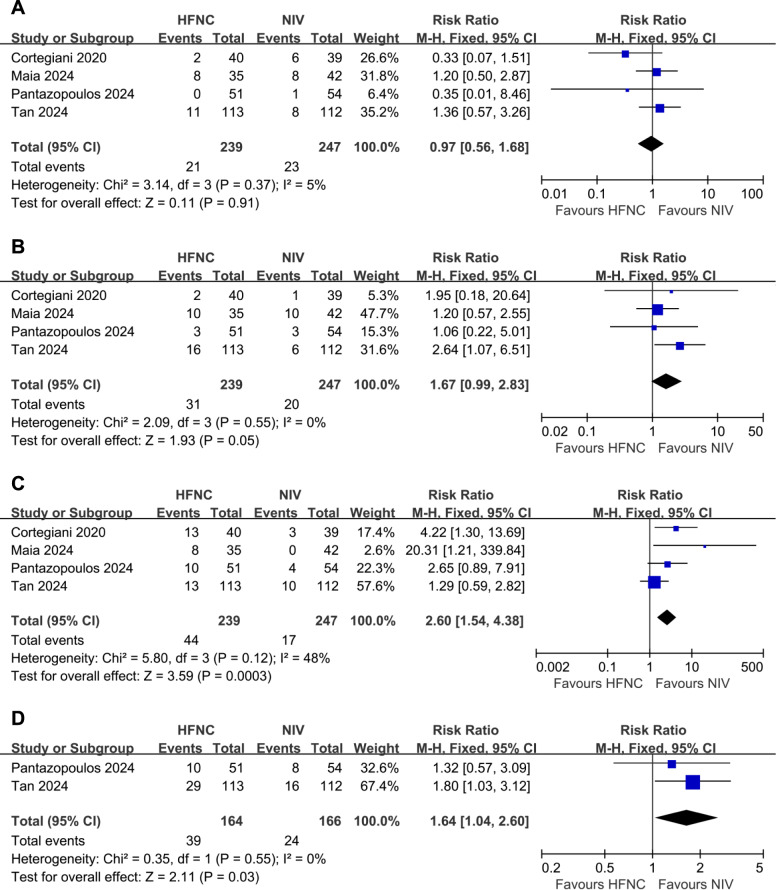
Forest plot assessing the efficacy of HFNC versus NIV on **A** all-cause mortality, **B** intubation rates, **C** treatment switch rates, and **D** treatment failure rates

Four RCTs reported data on all-cause death (486 patients). There was no statistically significant difference in all-cause mortality between the two groups (RR 0.97, 95% CI 0.56 to 1.68; P = 0.91 Fig. 3A). There was no significant heterogeneity (I^2^ = 5%; P = 0.37). The all-cause mortality in HFNC groups was 8.79% compared with 9.31% in NIV groups.

### Tracheal intubation

Data on patients with tracheal intubation were extracted from four RCTs (486 patients). The intubation rate did not differ significantly between the two groups (RR 1.67, 95% CI 0.99 to 2.83; P = 0.05 Fig. 3B), with a rate of 12.97% versus 8.10%. There was no significant heterogeneity (I^2^ = 0%; P = 0.55).

### Treatment switch

Data on patients with treatment switch were available from four randomized trials (486 patients). The treatment switch rate was statistically higher in HFNC groups (RR 2.60, 95% CI 1.54 to 4.38; P = 0.0003 Fig. 3C). There was no significant heterogeneity (I^2^ = 48%; P = 0.12). The treatment switch rate in HFNC groups was 18.41% compared with 6.88% in NIV groups.

### Treatment failure

Two RCTs reported data on treatment failure (330 patients). The treatment failure rate was significantly higher in HFNC groups (RR 1.64, 95% CI 1.04 to 2.60; P = 0.03 Fig. 3D). There was no significant heterogeneity (I^2^ = 0%; P = 0.55). The treatment failure rate in HFNC groups was 23.78% compared with 14.46% in NIV groups.

## Discussion

The aim of this meta-analysis was to compare the efficacy of HFNC and NIV in patients with AECOPD and hypercapnic ARF. Based on the current results, we observed that treatment switch and treatment failure rates were significantly higher in HFNC groups. Nearly one-fifth of the patients in the HFNC group were switched to NIV, which might be an important reason for the non-inferiority of HFNC to NIV.

COPD with its comorbidities is one of the five major causes of death worldwide [14]. AECOPD have a significant impact on individual patients and healthcare systems, and they are the largest component of the socioeconomic burden of COPD. For patients with AECOPD whose arterial blood pH is between 7.25 and 7.35 and without a metabolic cause for acidosis, intubation and mechanical ventilation are usually not considered necessary. It is precisely in this group of patients that there is the strongest evidence base to support the use of bilevel non-invasive ventilation [3]. Bilevel NIV can relieve the feeling of dyspnea, reduce the need for immediate intubation, shorten the length of stay in the intensive care unit (ICU), and improve survival rates.

HFNC is a respiratory support device. It is used during the early non-invasive management stage of ARF, working together with conventional oxygen therapy (COT) and NIV. HFNC has benefits in both clinical (such as high patient comfort and ease of use) and physiological (such as high oxygenation, alveolar recruitment, humidification and heating, increased secretion clearance, and reduction of dead space) aspects [15]. These benefits can prevent the deterioration of lung function and avoid tracheal intubation [16, 17]. However, there is limited evidence regarding the most appropriate form of non-invasive respiratory support in the context of hypercapnic ARF. Compared with COT and NIV, HFNC offers greater comfort and is better tolerated by patients. However, its ability to relieve the load on respiratory muscles in ARF may be lower than that of NIV [7]. For patients who do not respond well to treatment with HFNC or NIV, prolonging the duration of non-invasive respiratory support may lead to a delay in intubation and an increase in in-hospital mortality [18, 19]. NIV (alternating with HFNC) may be a better choice. In mechanically ventilated patients at high risk of extubation failure, the use of HFNC with NIV after extubation significantly reduced the risk of reintubation compared with HFNC alone [20]. The use of NIV + HFNC in patients with hypercapnic ARF needs further investigation.

This study has some differences from previous ones. Previous meta-analyses either focused on the changes in arterial blood gas analysis indicators [6], or included patients post-extubation [6, 21, 22], or incorporated non-COPD patients [23]. HFNC has better comfort and tolerability. However, the treatment switch rate in the HFNC group is about three times that of the NIV group in this meta-analysis. This may mask the fact that HFNC might be less effective than NIV, as reflected by the difference in the treatment failure and treatment switch rates between the two groups.

This study met most of the methodological criteria recommended for systematic reviews and meta-analyses [24]. However, some limitations need to be considered when interpreting the results of this study. Firstly, the number of included studies was small, which may have reduced the power of the results. Secondly, the parameter settings of HFNC and NIV varied between studies. Thirdly, all included studies were unblinded due to the treatments utilizing clearly different devices. Fourthly, the severity of hypercapnic acidosis in terms of PaCO_2_ and/or pH varied between studies. Lastly, this meta-analysis was not patient-level, so the results should be considered provisional.

## Conclusions

Compared with NIV, HFNC was not associated with increased mortality and intubation rate. More patients receiving HFNC oxygen therapy experienced treatment failure and switched to NIV, which may mask the fact that HFNC is inferior to NIV in patients with AECOPD and hypercapnic ARF. Given the limitations of the evidence we found, further large-scale, high-quality studies are needed.

## Supplementary Information

Supplementary material 1

## Data Availability

Extracted data are available on request to the corresponding author.
