# Supplementary material for: High flow nasal therapy versus noninvasive ventilation for AECOPD with acute hypercapnic respiratory failure: a meta-analysis of randomized controlled trials
Source: Ann Intensive Care. 2025 May 14;15:64. doi: 10.1186/s13613-025-01480-w (PMC12075079; doi:10.1186/s13613-025-01480-w)
Supplement: Supplementary file 1 — Supplementary material 1 [file 13613_2025_1480_MOESM1_ESM.docx]

**Table S1** Search strategies for all databases

**Table A** Search strategy in PubMed

| **Search strings** | **Items found** |
| --- | --- |
| (“high flow” OR “HFNC”) AND (“chronic obstructive pulmonary disease” OR “COPD”) AND (“hypercapnic” OR “acidosis” OR “hypercapnia” OR “acidotic”) | 124 |

**Table B** Search strategy in EMBASE

| **Search strings** | **Items found** |
| --- | --- |
| ('high flow' OR 'hfnc') AND ('chronic obstructive pulmonary disease'/exp OR 'chronic obstructive pulmonary disease' OR 'copd'/exp OR 'copd') AND ('hypercapnic' OR 'acidosis'/exp OR 'acidosis' OR 'hypercapnia'/exp OR 'hypercapnia' OR 'acidotic') | 315 |

**Table C** Search strategy in Cochrane Library

| **Search strings** | **Items found** |
| --- | --- |
| (“high flow” OR “HFNC”) AND (“chronic obstructive pulmonary disease” OR “COPD”) AND (“hypercapnic” OR “acidosis” OR “hypercapnia” OR “acidotic”) | 100 |

**Table D** Search strategy in clinicaltrials.gov

| **Status** | **Condition/disease** | **Intervention/treatment** | **Other terms** | **Items found** |
| --- | --- | --- | --- | --- |
| All studies | (“chronic obstructive pulmonary disease” OR “COPD”) | (“high flow” OR “HFNC”) | (“hypercapnic” OR “acidosis” OR “hypercapnia” OR “acidotic”) | 40 |
